# High Cortisol and the Risk of Dementia and Alzheimer’s Disease: A Review of the Literature

**DOI:** 10.3389/fnagi.2019.00043

**Published:** 2019-03-01

**Authors:** Sami Ouanes, Julius Popp

**Affiliations:** ^1^Department of Psychiatry, Hospital of Cery, University Hospital of Lausanne, Lausanne, Switzerland; ^2^Department of Psychiatry, Hamad Medical Corporation, Doha, Qatar; ^3^Geriatric Psychiatry, Department of Mental Health and Psychiatry, Geneva University Hospitals, Geneva, Switzerland

**Keywords:** cognition, cortisol, memory, executive functions, dementia

## Abstract

**Introduction:** Cortisol effects on the brain are exerted through two distinct receptors, inducing complex and even opposite effects on the cerebral structures implicated in the various cognitive functions. High cortisol may also have deleterious effects on the brain structures and contribute to neurodegeneration, in particular Alzheimer’s disease (AD), via different mechanisms.

**Objective:** To examine the interrelationships between cortisol, cognitive impairment and AD.

**Methods:** Review of the literature.

**Results:** Clinical studies found that elevated cortisol was associated with poorer overall cognitive functioning, as well as with poorer episodic memory, executive functioning, language, spatial memory, processing speed, and social cognition; while in animals, glucocorticoid administration resulted in cognitive impairment and abnormal behavior. In cognitively healthy subjects, higher cortisol levels have been associated with an increased risk of cognitive decline and AD. Subjects with dementia and Mild Cognitive Impairment (MCI) due to AD have been found to have higher CSF cortisol levels than cognitively healthy controls. Elevated CSF cortisol may also be associated with a more rapid cognitive decline in MCI due to AD. Elevated cortisol levels have been also found in delirium. High cortisol may mediate the impact of stressful life events, high neuroticism, depression, sleep disturbances, as well as cardiovascular risk factors on cognitive performance, neurodegeneration, and cognitive decline. High cortisol may also exert neurotoxic effects on the hippocampus, and promote oxidative stress and amyloid β peptide toxicity. Further possible underlying mechanisms include the interactions of cortisol with inflammatory mediators, neurotransmitters, and growth factors.

**Conclusion:** Elevated cortisol levels may exert detrimental effects on cognition and contribute to AD pathology. Further studies are needed to investigate cortisol-reducing and glucocorticoidreceptor modulating interventions to prevent cognitive decline.

## Introduction

Corticosteroids seem to be among the hormones with the most important effects on the brain function. Indeed, corticosteroids have been associated with effects on mood, stress, anxiety, sleep, appetite, as well as cognition (Lupien et al., 2007; Wolkowitz et al., 2009; Copinschi and Caufriez, 2013).

Once released from the adrenal cortex, cortisol, the main glucocorticoid in humans, easily crosses the blood–brain barrier, owing to its lipophilic character (Wolkowitz et al., 2009). Cortisol binds to specific intracellular receptors in the brain, in particular in regions implicated in cognitive functions (McEwen, 2007; Daskalakis et al., 2013; Vogel et al., 2016). Once activated, these receptors bind to “ hormone response elements” in the DNA and regulate the transcription of target genes (Joels, 2006).

The resulting effects on cognition seem to be complex and involve several cognitive domains (Lupien et al., 2007; Lee C.M. et al., 2008; Tatomir et al., 2014; Geerlings et al., 2015; Vogel et al., 2016). Different levels of cortisol likely produce different and even sometimes opposite effects (de Kloet et al., 1999; Joels, 2006). While some of these effects are acute (Lupien and McEwen, 1997; Lupien et al., 2002; Meir Drexler and Wolf, 2016), some appear to be long-lasting and may even involve long-term changes in the brain structure (Geerlings et al., 2015).

Altered Hypothalamic-Pituitary-Adrenal (HPA) axis functioning, and in particular high cortisol levels in the elderly have been associated with an increased risk for dementia and Alzheimer’s disease (AD) (Lupien et al., 1999; Rothman and Mattson, 2010; Ennis et al., 2017; Notarianni, 2017).

A better understanding of these interrelationships between cortisol, cognition and dementia may open the door to new prevention and therapeutic options involving the HPA axis. The effects of cortisol on emotional memory had already led to therapeutic trials of corticosteroids and corticosteroid receptor antagonists/modulators in AD (Pineau et al., 2016), as well as in treating or preventing post-traumatic stress disorder (PTSD) (Daskalakis et al., 2013), as well as in treating depression (Wolkowitz and Reus, 1999; Kling et al., 2009).

## Glucocorticoid Receptors and Cortisol Effects on Cognition

Cortisol exerts its effects on cognition through two types of receptors: type I (Mineralocorticoid Receptors, MRs) and type II (Glucocorticoid Receptors, GRs) (Joels, 2006; Daskalakis et al., 2013). Surprisingly, the MRs display 6 to 10 times higher affinity for glucocorticoids, mainly cortisol, than GRs (de Kloet et al., 1999; Joels, 2006).

These receptors are expressed differently throughout the brain. Indeed, the hippocampus, mainly implicated in episodic memory, expresses both MRs and GRs, whilst the prefrontal cortex, primarily responsible for executive functions, only expresses GRs (Lupien et al., 2007; McEwen, 2007). While MRs have been associated with positive/enhancing effects on the cognitive performance, GRs have, on the contrary, been linked to negative inhibitory effects. In this regard, it has been found that infusion of a GR antagonist, but not of MR antagonist, in the medial prefrontal cortex of a mouse blocked the deleterious effects of glucocorticoids on working memory (Barsegyan et al., 2010).

**Figure 1:**
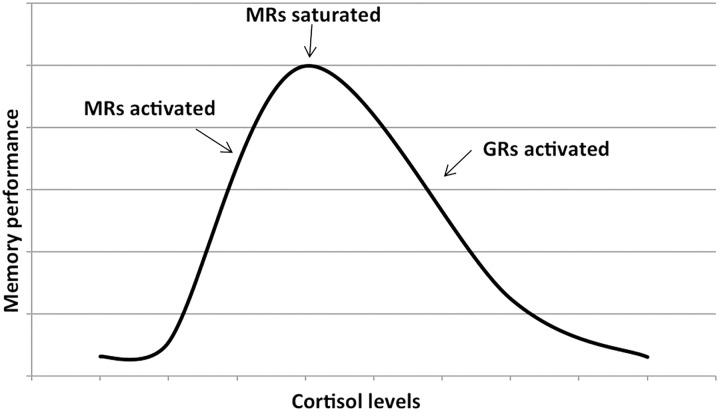
Dose–response relationship between the memory performance and the cortisol levels. The first part of the plot shows that memory performance increases as cortisol levels increase (due to the activation of mineralocorticoid receptors or MRs). As soon as the MRs are saturated, further increase in cortisol levels activates the glucocorticoids receptors or GRs and memory performance decreases. Adapted with permission from Lupien et al. (2007).

**Figure 2:**
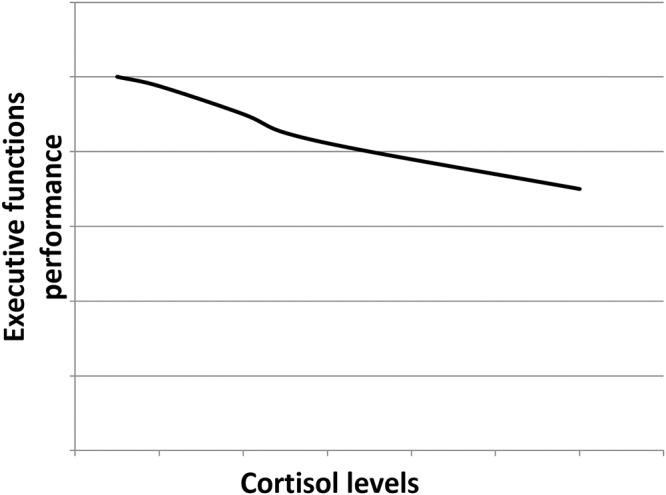
Hypothetical dose–response relationship between the executive functions performance and the cortisol levels. As the prefrontal cortex only expresses GRs, the higher the cortisol levels, the poorer the executive functions performance.

Cortisol effects on the hippocampus-related cognitive performance have often been described by the means of an inverted-U shape plot (Figure 1). Indeed, in the hippocampus, where both GRs and MRs are expressed, moderate levels of cortisol only activate the receptors with the higher affinity, i.e., MRs, leading to memory enhancement effects. As cortisol levels increase, this positive effect increases till MRs are saturated. Starting from this point, as cortisol levels rise, GRs are increasingly activated thus leading to increasingly detrimental effects on the memory. Distinctly, the effects of cortisol on executive functions are likely more linear. Since the prefrontal cortex region, mostly responsible for executive functions, only expresses GRs, higher levels of cortisol may lead to worsened executive functioning (Figure 2; Lupien et al., 2007; McEwen, 2007). The fact that both adrenal insufficiency and Cushing’s disease have been associated with impaired declarative memory (Forget et al., 2016; Tiemensma et al., 2016) bolsters this biphasic effect hypothesis. Another argument is that the administration of the MR agonist fludrocortisone has been found to improve verbal and visuospatial memory performance in young as well as elderly healthy subjects (Hinkelmann et al., 2015); whereas the administration of hydrocortisone (mimicking the endogenous cortisol effects on both GRs and MRs) has been shown to enhance at lower doses yet impair at higher doses verbal memory retrieval in healthy subjects (Domes et al., 2005).

These effects that cortisol exerts on the brain structures involved in cognition are possibly mediated by modifications in responses to serotonin, in β-adrenergic receptor activation, in calcium influx, as well as in long-term potentiation (LTP), a process referring to a long-term strengthening of synaptic connections contributing to memory formation and consolidation (Joels, 2006; Lupien et al., 2007). Indeed, GR activation facilitates β-adrenergic signaling thus leading to the formation of adenosine 3′,5′-cyclic monophosphate (cAMP) and cAMP-dependent protein kinase (PKA). This pathway, once activated, is thought to inhibit the medial prefrontal cortex thus leading to an impairment in frontal functions, in particular working memory (McGaugh and Roozendaal, 2002; Barsegyan et al., 2010). Glucocorticoids have also been found to display certain effects on the hippocampus via actions on the serotoninergic system following the same biphasic pattern as described in Figure 1. Indeed, glucocorticoids can promote via MRs and, at the same time, inhibit via GRs the 5HT1A activation in hippocampal CA1 pyramidal cells (de Kloet et al., 2018).

Moreover, glucocorticoids have been reported to alter LTP in opposite directions depending on the MR/GR activation ratio: when the MR/GR activation ratio is high (central part of the inverted U-shaped plot, Figure 1), LTP is enhanced thus improving long-term memory consolidation. On the contrary, when the MR/GR ratio is low (extremes of the inverted U-shaped plot, Figure 1), LTP is suppressed thus worsening long-term memory consolidation (de Kloet et al., 1999; Lupien et al., 2007).

In clinical studies, most studies examining the link between cortisol levels and global cognitive performance among non-demented older adults found that higher cortisol levels have been associated with poorer overall cognitive performance (Lupien et al., 2007; Lee B.K. et al., 2008; Ouanes et al., 2017a; Sang et al., 2018). Likewise, most (Beluche et al., 2010; Geerlings et al., 2015; Segerstrom et al., 2016; Ouanes et al., 2017a,b; Echouffo-Tcheugui et al., 2018), even though not all (Lee B.K. et al., 2008) studies exploring the relationship between episodic memory and cortisol levels have found an association between elevated cortisol and poorer episodic memory among older adults without dementia. These findings suggest that, even at levels that are within the normal range, cortisol can still activate GRs, and not just MRs. This also suggests that relatively small differences in cortisol levels can exhibit significant effects on memory performance. Studies exploring the relationship between cortisol levels and prefrontal cortex-mediated cognitive functions, mainly executive functions, processing speed and working memory, have found more discrepant results: while the expected negative association was reported in certain studies (Lee B.K. et al., 2008; Beluche et al., 2010; Geerlings et al., 2015), other studies failed to find such an association (Ouanes et al., 2017a,b; Echouffo-Tcheugui et al., 2018).

The differences in populations, assessment tools, as well as the likely effects of possible confounding factors including age, gender, educational level, as well as other neuroendocrine and psychological factors might explain these discrepancies.

## Cortisol and Cerebral Structural Changes

High cortisol has also been linked to decreased volume of several brain regions involved in cognitive functions. In fact, in a study by Geerlings et al. (2015) involving 4244 non-demented subjects, elevated evening cortisol was found to be associated with decreased volumes in all brain regions, in particular the gray matter (Geerlings et al., 2015). Similar findings have been reported in the dementia-free Framingham Heart Study participants: elevated cortisol was associated with decreased total brain volume, in particular decreased occipital and frontal gray matter volumes. In the same study, increased cortisol levels were associated with some microstructural changes, specifically in the corpus callosum and the posterior corona radiate (Echouffo-Tcheugui et al., 2018).

**Figure 3:**
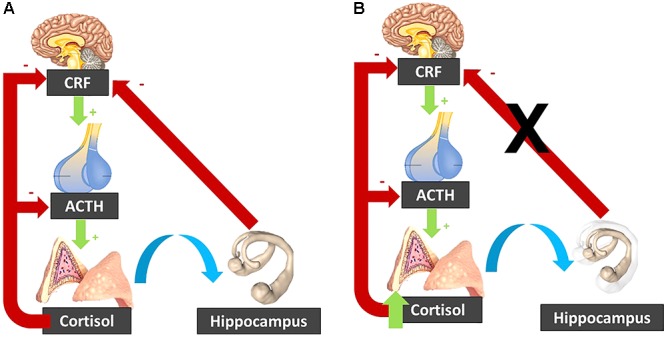
**(A)** In normal circumstances, the CRF released by the hypothalamus activates ACTH release by the pituitary gland, which stimulates the adrenal glands to secrete cortisol. Cortisol inhibits its own secretion via a negative feedback loop. The hippocampus inhibits the hypothalamo-pituitary-adrenal axis. **(B)** When cortisol is elevated, it can induce hippocampal atrophy, which “lifts the brake” on the hypothalamo-pituitary-adrenal axis. The resulting cortisol increase induces further hippocampal atrophy, resulting in a vicious circle. CRF, corticotropin-releasing factor; ACTH, Adrenocorticotropic hormone.

In addition, high levels of cortisol have been linked to hippocampal atrophy (Tatomir et al., 2014). This atrophy can be the consequence of the exposure to increased cortisol levels. Indeed, in Cushing’s disease, the observed hippocampal atrophy is reversed following treatment and normalization of the cortisol levels (Starkman et al., 1999). Yet, this atrophy can also be a cause of the elevated cortisol levels. Indeed, the hippocampus exerts an inhibitory effect on the HPA axis activity, and hence hippocampal atrophy might disinhibit the HPA axis leading to increased cortisol (Geerlings et al., 2015; Figure 3).

These effects on the hippocampal volume may be partly due to changes in brain-derived neurotrophic factor (BDNF) expression in the hippocampus (Suri and Vaidya, 2013). Similarly to their effects on cognitive performance, MR activation seems to increase whereas GR activation seems to decrease BDNF expression in the hippocampus (Kino et al., 2010; Suri and Vaidya, 2013).

In a study by Cox et al. (2015) elevated salivary cortisol levels at the start and at the end of a cognitive task appointment have been associated with a poorer white matter structure, i.e., greater white matter hyperintensity volume and/or elevated general factor of tract mean diffusivity. These findings suggest that aside from the “acute effects” of cortisol on cognition, chronically elevated cortisol levels likely bring about brain structural changes that may reflect long-term cognitive deficits.

## HPA-Axis Dysregulation, Cortisol and Alzheimer’s Disease Pathology, Disease Risk and Clinical Course

Glucocorticoids have been reported to promote oxidative stress and to increase amyloid β (Aβ) peptide toxicity in cultured hippocampal neurons (Goodman et al., 1996). Besides, in a mouse model of AD, elevated cortisol has been linked to exacerbated Aβ peptide and tau pathology in the brain (Green et al., 2006).

In primates, year-long high-dose exposure to glucocorticoids was associated with decreased insulin-degrading enzyme levels, a candidate protease for the clearance of Aβ in the brain. At the same time, the Aβ1-42/Aβ1-40 ratio was increased indicating a relative shift toward increased production of the more brain toxic Aβ1-42 (Kulstad et al., 2005).

In a cross-sectional study examining the links between cardiovascular risk factors and Aβ brain burden as determined by Pittsburgh Compound B-positron emission tomography (PiB-PET), an association has been found between plasma cortisol and Aβ brain burden (Toledo et al., 2012).

Together, these findings suggest that increased cortisol may induce and/or exacerbate cerebral AD pathology by increasing Aβ brain burden, tau pathology as well as oxidative stress, which can all contribute to neurodegeneration. Effects on Aβ likely entail decreasing Aβ clearance as well as promoting the cleavage of Aβ into the most toxic compound (Aβ1-42) (Kulstad et al., 2005).

The aforementioned cerebral changes associated with elevated cortisol likely translate into findings of associations between increased cortisol levels and clinical features of AD, as shown in several clinical studies that reported increased cortisol levels in patients with clinical AD dementia (Dong and Csernansky, 2009; Popp et al., 2009; Ennis et al., 2017), and cortisol levels have even been found to correlate with the severity of the cognitive impairment (Pedersen et al., 2001; Zverova et al., 2013).

A prospective study by Ennis et al. (2017) S found that elevated cortisol (as measured by the urinary free cortisol/creatinine ratio) and elevated intra-subject cortisol variability (as measured by the within-person urinary free cortisol/creatinine ratio variability) were associated with a 1.31- and 1.38-times increase in AD risk. Furthermore, in cognitively healthy older adults with Aβ positive PET imaging, high cortisol levels have been found to be associated with a faster decline in global cognition, in episodic memory, as well as in executive functioning, independently of age, sex, APOE genotype, or anxiety symptoms (Pietrzak et al., 2017). In a population based cohort study in 537 non-demented older adults (65 years or more at baseline), we found salivary cortisol day profiles to be not associated with faster cognitive decline over an average 5.3 years. However, preliminary analysis suggests that higher morning salivary cortisol measures may be associated with slight decline in global cognition (Albanese et al., 2018).

Higher plasma cortisol levels in patients with AD dementia have been associated with a more rapid cognitive decline in some studies (Pedersen et al., 2001; Huang et al., 2009). Similarly, Csernansky et al. (2006) found that high plasma cortisol levels were associated with faster cognitive decline in individuals with very mild or mild AD dementia.

In a cohort study from the Alzheimer Disease Neuroimaging Initiative (ADNI) investigating biomarkers able to predict progression from mild cognitive impairment (MCI) to AD within 1–6 years, plasma cortisol was one of the six biomarkers found to provide an accurate prediction (Lehallier et al., 2016).

Moreover, cortisol concentrations in the cerebrospinal fluid (CSF) have been found to be higher in subjects with dementia and MCI due to AD compared to control subjects (Popp et al., 2009, 2015). In MCI due to AD, high CSF cortisol was also predictive of a more rapid cognitive decline (Popp et al., 2015). Hence, elevated cortisol appears to contribute to exacerbate AD brain pathology, thereby contributing to the disease progression both pathologically and clinically. Cortisol levels appear to be increased at early stages of AD, and fasting plasma and CSF cortisol levels may even be pre-clinical markers (Notarianni, 2017).

These findings may be explained by increased Aβ neurotoxicity related to higher cortisol levels as well as neurodegeneration and functional impairment of the hippocampus occurring early in the course of the disease as both a consequence of exposure to high cortisol levels and a cause of HPA axis disinhibition, hence a vicious circle (Geerlings et al., 2015).

## Cortisol, Cognition and Mediating Factors

Certain factors may further explain the links between cortisol, cognitive impairment and dementia. Indeed, some of these factors may bring about HPA axis alterations that could affect cognition and the risk for dementia, in particular AD. These factors may include life events (Ouanes et al., 2017a), personality (Ouanes et al., 2017b; Tautvydaite et al., 2017; Terracciano et al., 2017), sleep disorders (Haba-Rubio et al., 2017), depression (Salvat-Pujol et al., 2017).

At the same time, some other factors such as metabolic syndrome, insulin resistance and effects on inflammation may mediate the effects of cortisol on cognition and brain structural changes (Kim and Feldman, 2015; Martocchia et al., 2016).

### Cortisol, Cognition, Trauma and Life Events

Early trauma (of physical, sexual or emotional nature) has been linked to long-term cognitive deficits in adulthood (consisting in impaired spatial working memory and pattern recognition memory) in a study by Majer et al. (2010); however, this finding was not replicated in other studies (Saigh et al., 2006). Early stress has also been shown to be associated with structural and functional changes in brain regions involved in cognitive functions, including the frontal cortex as well as the hippocampus (Lupien et al., 2009).

Early trauma is also one of the most important established risk factors for PTSD. PTSD has been shown to be associated with an increased risk of dementia in both genders over an average of 8 years of follow-up (hazard ratio: 1.73[1.47, 2.02]) (Flatt et al., 2017). Nevertheless, Burri et al. (2013) found that the long-term cognitive deficits associated with PTSD were likely independent of earlier childhood adversity.

Aside from early trauma and PTSD, stressful life events have often been associated with HPA activation. Yet, some studies, conversely, showed decreased cortisol following stressful life events (Miller et al., 2007; Daskalakis et al., 2013). Results of the studies exploring the relationship between life events and cognition have been discrepant. On the one hand, several studies highlighted associations between stressful events and poorer subsequent cognitive performance, in particular in memory and executive functions (Xavier et al., 2002; VonDras et al., 2005; van Gelder et al., 2006; Lupien et al., 2007) above and beyond the impact of depression (Comijs et al., 2011). Importantly, stressful events have been also associated with an elevated risk of late-life dementia (Johansson et al., 2010) and late-life cerebral atrophy, and white matter lesions (Johansson et al., 2012). On the other hand, other studies failed to find any association between stressful life events and cognitive performance in the elderly (Ward et al., 2007; Fountoulakis et al., 2011; Sundstrom et al., 2014) and some even showed a possible improvement in cognition following certain stressful events (Deeg et al., 2005).

In a study exploring the mediation hypothesis between cortisol, life events and cognition in 796 non-demented subjects aged at least 65 we found elevated salivary cortisol levels to be linked to poorer cognitive performance, but this association was not related to life events (Ouanes et al., 2017a).

These discrepancies regarding the relationships between life events and cortisol on the one hand, and life events and cognition on the other hand, may be explained by different life events displaying different effects on cortisol and thus on cognition (Ouanes et al., 2017a).

### Cortisol, Cognition and Personality

High neuroticism is the personality trait most consistently often associated with high cortisol (Bridges and Jones, 1968; van Eck et al., 1996; Miller et al., 1999, 2016; Portella et al., 2005; Yoshino et al., 2005; Gerritsen et al., 2009; Nater et al., 2010; Garcia-Banda et al., 2014). However, other studies found no association (Adler et al., 1997; Schommer et al., 1999; Ferguson, 2008), or even an opposite link (Ballenger et al., 1983; LeBlanc and Ducharme, 2005).

Higher neuroticism has also been reported to be cross-sectionally linked to lower cognitive performance above and beyond the effects of depression (Jorm et al., 1993; Boyle et al., 2010), especially to poorer episodic memory (Jorm et al., 1993; Meier et al., 2002; Klaming et al., 2016). In addition, high neuroticism scores have been found in association with elevated risk of AD (Terracciano et al., 2014).

A few studies examined the relationship between the other personality traits and cognitive performance and risk of dementia. Lower pre-morbid conscientiousness, agreeableness, openness and extraversion have been associated, although not consistently, with lower cognitive performance and higher risk for AD (Terracciano et al., 2014, 2017; Tautvydaite et al., 2017). In a cohort of memory clinic patients and cognitively healthy elderly volunteers we found lower extraversion and openness to correlate with CSF markers of AD pathology: tau, ptau-181, tau/Aβ1–42, and ptau-181/Aβ1–42 ratios, but not with the Aβ1–42 level (Tautvydaite et al., 2017).

In a population-based cohort study examining the interrelationships between cortisol, cognition and personality traits, salivary cortisol did not seem to mediate the link between personality traits and cognitive deficits (Ouanes et al., 2017b).

Besides methodological differences, these observed discrepancies may be due to the impact of depression and/or anxiety which has been controlled for in a few studies but not in others, but also to the difficulties (in cross-sectional studies, mainly) to disentangle pre-morbid personality traits from the personality modifications accompanying the cognitive decline.

### Cortisol, Cognition and Sleep Disorders

Cognitive impairment has been associated with more time spent in stage N1 (first step of non-rapid eye movement sleep) and less in stage N3 (third step of non-rapid eye movement sleep) and in REM sleep, lower sleep efficiency, and more wake after sleep onset, as well as more severe sleep disordered breathing (as evidenced by higher apnea/hypopnea index or AHI, and higher oxygen desaturation index or ODI) (Haba-Rubio et al., 2017).

In a study of the same research group, involving 456 elderly non-demented subjects, obstructive sleep apnea (OSA) has been found to be linked to cognitive impairment, but the relationship did not appear to be mediated by diurnal cortisol levels (Haba-Rubio et al., 2018).

In other studies, OSA has been associated with increased nocturnal plasma cortisol levels (Chopra et al., 2017). Edwards et al. (2014) found that higher night-time cortisol was associated with worse cognitive performance, mainly affecting memory, above and beyond the apnea severity in a sample of patients with OSA.

Taken together, the results of these studies highlight links between cognitive performance and sleep disorders on the one hand, and between cortisol levels and cognitive functioning on the other hand, but do not provide evidence to support that cortisol may actually mediate the relationship between sleep disorders and cognitive impairment.

### Cortisol, Cognition and Depression

Depression has been associated, on the one hand with HPA axis hyperactivity and impaired negative feedback (Anacker et al., 2011), and on the other hand with cognitive deficits involving attention, episodic memory and executive functions (Salvat-Pujol et al., 2017). Depression has also been tied to late-life dementia, in particular with vascular and AD dementia (Brunnstrom et al., 2013). This association is not just a mere comorbidity, as late-life depression may also be a risk factor for both AD and vascular dementia (Diniz et al., 2013; Herbert and Lucassen, 2016). In the AGES-Reykjavik population-based study (Geerlings et al., 2017), both current major depressive disorder and high evening cortisol levels were associated with an higher risk of incident AD and non-AD dementia, but cortisol did not seem to be a major factor explaining the relation between depression and risk of dementia.

Some of the observed cognitive deficits in verbal and visual memory and executive functions may remain present even after the depressive symptoms fully remitted (Herrera-Guzman et al., 2010; Rock et al., 2014; Salvat-Pujol et al., 2017). Likewise, the HPA axis abnormalities associated with depression may persist even after remission (Lok et al., 2012; Salvat-Pujol et al., 2017), possibly constituting trait rather than state markers for depression, even though this remains a matter of debate (Zverova et al., 2013; Salvat-Pujol et al., 2017).

Remission status in depression did not moderate the association between cognitive performance and the Dexamethasone suppression test ratio or the cortisol awakening response (CAR), defined by the increase in cortisol secretion after awakening (Fries et al., 2009). However, remission appeared to moderate the association between cortisol slope defined by the difference between maximal and minimal cortisol levels during the nyctemera, and certain cognitive tasks assessing processing speed and executive function (Salvat-Pujol et al., 2017). HPA axis alteration in depression may inhibit neurogenesis, partly through reducing BDNF which is involved in hippocampal neurogenesis, thus possibly explaining one of the mechanisms by which depression may be a risk factor for AD (Herbert and Lucassen, 2016).

Delirium is common in AD, and it is associated with more rapid clinical disease progression (Popp, 2013). Depression symptoms and cognitive impairment have been independently associated with higher risk of developing delirium. In a yet-to be-published study by the same team, increased cortisol levels have been observed in patients with delirium suggesting HPA axis dysregulation to be involved in the pathophysiology of delirium. In a cohort of elderly patients undergoing elective cardiac surgery, pre-operative geriatric depression scale scores were found to predict post-operative delirium. However, pre-operative morning plasma cortisol levels were not associated with post-operative delirium in this study.

Whether and how HPA axis dysregulation and increased cortisol levels may contribute to the magnitude of cognitive and non-cognitive symptoms in AD, needs further investigation. In a study in patients with AD dementia, plasma cortisol levels have been shown to reflect the degree of cognitive deficits in AD dementia rather than the severity of the comorbid depression (Zverova et al., 2013).

Altogether, these data suggest that, mostly, cognitive deficits linked to increased cortisol and HPA axis dysregulation cannot be entirely explained by a co-occurring depressive disorder. AD pathology may exacerbate HPA axis dysregulation which may contribute to the manifestation of depressive symptoms and to the severity of cognitive impairment and increase the risk of other non-cognitive syndromes, including delirium. Even though depression-associated HPA axis dysregulation may predispose to and/or exacerbate the course of AD (Herbert and Lucassen, 2016), studies suggest a link between HPA axis dysregulation and AD itself above and beyond depression.

### Cortisol, Cognition and the Metabolic Syndrome

Elevated cortisol levels have been tied to insulin resistance and metabolic syndrome, which in turn, have been associated with both AD and vascular dementia. (Kim and Feldman, 2015; Martocchia et al., 2016). Hence, elevated cortisol may lead, through its metabolic syndrome-associated effects on glucose, blood pressure and lipids, to an increased cardiovascular risk (Lattanzi and Silvestrini, 2017). Indeed, higher cortisol has been associated with a higher number of carotid plaques (Hamer et al., 2010). The resulting vascular lesions in the brain may directly induce cognitive disturbances, but can also contribute to the neurodegeneration observed in AD (Attems and Jellinger, 2014). Moreover, insulin resistance itself may negatively influence the amyloid cascade (Stefanelli et al., 2014).

At the same time, AD-associated hypercortisolemia, present at very early stages, may also induce pre-diabetes. The resulting increased insulin secretion can further exacerbate the hypercortisolemia, thus possibly negatively affecting the course of AD (Notarianni, 2017).

### Cortisol, Cognition and Inflammation

While cortisol is generally known to exert broad anti-inflammatory effects, high cortisol levels may activate NACHT, LRR and PYD domains-containing protein 1 (NLRP-1) inflammasome in hippocampal neurons, thus promoting neuroinflammation and thereby neuronal injury (Zhang et al., 2017).

Moreover, certain cytokines, in particular IL-1-Beta and IL-6, which are also known to be involved in the pathophysiology of AD, can activate the HPA axis (Besedovsky and del Rey, 2000). The resulting increased cortisol can reinforce the toxic effects on the hippocampus exerted by the pro-inflammatory cytokines (Sudheimer et al., 2014), thus contributing to the pathophysiology of AD.

## Cortisol and Potential Preventive and Therapeutic Interventions for Alzheimer’s Disease:

Since increased cortisol has been associated with both AD pathology and more rapid clinical disease progression, and since most detrimental effects of cortisol are likely exerted via GRs, therapeutic interventions targeting the GRs have been investigated. Indeed, the GR antagonist mifepristone has been shown to decrease both Aβ and tau load in the brain as well as to improve the pathologically induced cognitive impairments in a triple-transgenic (3xTg AD) mouse model of AD (Baglietto-Vargas et al., 2013). In a similar way, mifepristone has been shown to reduce the hippocampal Aβ levels and rescue the cognitive deficits induced by early life stress in APP/PS1 transgenic mice (Lesuis et al., 2018). As these pathological processes start years or even decades before the onset of the first symptoms, cortisol lowering or cortisol effects modulating interventions in midlife may slow down the development of amyloid pathology and neurodegeneration, and prevent cognitive decline in later life (Lante et al., 2015; Lesuis et al., 2018). Such interventions could prove useful, in particular in subjects at risk for developing clinical AD (Pietrzak et al., 2017) and prone to stress, and HPA-axis dysregulation. Prevention trials with focus on cortisol or HPA-axis in human subjects with normal cognition have not been reported so far, however.

One randomized controlled trial in a small sample of patients with mild to moderate AD dementia showed improvement of cognitive performance in memory tasks, but the premature termination did not allow any firm conclusions regarding efficacy (Pomara et al., 2002). Other trials using mifepristone that were initiated were terminated without being published, indicating that these trials were not completed, or yielded negative results (O’Banion, 2013).

Also, several non-pharmacological intervention in subjects with MCI (Baker et al., 2010) or dementia (Woods et al., 2009; Schaub et al., 2018) have shown cortisol lowering effects.

However, there has been some loss of interest in GR antagonists because of their side effects due to GRs being ubiquitous, especially as more selective molecules, namely GR modulators have been developed (Canet et al., 2018).

Glucocorticoid Receptor modulators have been shown to normalize basal glucocorticoid plasma levels, decrease hippocampal Aβ peptide deposition, inhibit neuroinflammation, and apoptotic processes, and improve cognitive performance in a mouse model of AD (Pineau et al., 2016).

Another potential mechanism by which cortisol effects can be reduced pharmacologically is the inhibition of cortisol synthesis, one of the key enzymes being the 11β-hydroxysteroid dehydrogenase type 1 (11β-HSD1). Currently, a phase II trial of an 11β-HSD1 inhibitor (UE2343) as a potential treatment for AD is being conducted (Webster et al., 2017).

## Strengths and Limitations

This narrative review provides a concise overview of the different molecular, cellular, and clinical (including diagnostic, prognostic, and therapeutic) aspects of the interrelationships between cortisol, cognition, dementia, and AD. However, it does not cover all possible facets of these complex relationships. We focused on the most important and the most clinically relevant aspects of the topic, rather going in depth into one particular aspect of the topic.

## Conclusion

There is a growing body of evidence that increased cortisol may be deleterious for the late-life cognitive performance, and may be associated with an increased risk for cognitive decline and dementia, in particular dementia due to AD. In patients with AD, the increased cortisol at preclinical and early clinical stages is associated with a poorer prognosis and a more rapid cognitive decline. Increased cortisol may represent a pathophysiological mediator between stressful life events, personality, mood, and sleep, and may increase both the risk of AD and the extent of symptoms at clinical stages of the disease. Yet, the exact underlying mediating factors are not fully understood. Direct deleterious cortisol effects on the hippocampus and on the prefrontal cortex are likely, but also cortisol links with metabolic syndrome and neuroinflammation; and HPA axis disinhibition due to neurodegeneration are other possible mechanisms that may explain the association of cortisol with late-life cognitive impairment and AD.

Further studies are needed to confirm the value of cortisol levels as a possible preclinical marker associated with higher risk, and/or as a prognostic parameter in subjects with clinical AD. Future research may also bring in new HPA-based interventions for the prevention and/or management of symptoms, and of the clinical progression of AD.

## Author Contributions

SO participated in literature review and in writing the first draft of the manuscript. JP participated in literature review and revised the article.

## Conflict of Interest Statement

The authors declare that the research was conducted in the absence of any commercial or financial relationships that could be construed as a potential conflict of interest.

## References

[B1] Adler L. Wedekind D. Pilz J. Weniger G. Huether G. (1997). Endocrine correlates of personality traits: a comparison between emotionally stable and emotionally labile healthy young men. *Neuropsychobiology* 35 205–210. 10.1159/000119346 9246223

[B2] Albanese E. Preisig M. Castelao E. Ouanes S. Popp J. (2018). Salivary cortisol and 5y change in cognitive function in community dwelling, cognitively healthy older adults: the Psycolaus cohort study. *Alzheimers Dement.* 14:972 10.1016/j.jalz.2018.06.1304

[B3] Anacker C. Zunszain P. A. Carvalho L. A. Pariante C. M. (2011). The glucocorticoid receptor: pivot of depression and of antidepressant treatment? *Psychoneuroendocrinology* 36 415–425. 10.1016/j.psyneuen.2010.03.007 20399565 PMC3513407

[B4] Attems J. Jellinger K. A. (2014). The overlap between vascular disease and Alzheimer’s disease–lessons from pathology. *BMC Med.* 12:206. 10.1186/s12916-014-0206-2 25385447 PMC4226890

[B5] Baglietto-Vargas D. Medeiros R. Martinez-Coria H. LaFerla F. M. Green K. N. (2013). Mifepristone alters amyloid precursor protein processing to preclude amyloid beta and also reduces tau pathology. *Biol. Psychiatry* 74 357–366. 10.1016/j.biopsych.2012.12.003 23312564 PMC3633722

[B6] Baker L. D. Frank L. L. Foster-Schubert K. Green P. S. Wilkinson C. W. McTiernan A. (2010). Effects of aerobic exercise on mild cognitive impairment: a controlled trial. *Arch. Neurol.* 67 71–79. 10.1001/archneurol.2009.307 20065132 PMC3056436

[B7] Ballenger J. C. Post R. M. Jimerson D. C. Lake C. R. Murphy D. Zuckerman M. (1983). Biochemical correlates of personality traits in normals: an exploratory study. *Pers. Individ. Differ.* 4 615–625. 10.1016/0191-8869(83)90116-2

[B8] Barsegyan A. Mackenzie S. M. Kurose B. D. McGaugh J. L. Roozendaal B. (2010). Glucocorticoids in the prefrontal cortex enhance memory consolidation and impair working memory by a common neural mechanism. *Proc. Natl. Acad. Sci. U.S.A.* 107 16655–16660. 10.1073/pnas.1011975107 20810923 PMC2944727

[B9] Beluche I. Carriere I. Ritchie K. Ancelin M. L. (2010). A prospective study of diurnal cortisol and cognitive function in community-dwelling elderly people. *Psychol. Med.* 40 1039–1049. 10.1017/S0033291709991103 19814852 PMC2894868

[B10] Besedovsky H. O. del Rey A. (2000). The cytokine-HPA axis feed-back circuit. *Z. Rheumatol.* 59(Suppl. 2), II/26–30. 10.1007/s003930070014 11155800

[B11] Boyle L. L. Lyness J. M. Duberstein P. R. Karuza J. King D. A. Messing S. (2010). Trait neuroticism, depression, and cognitive function in older primary care patients. *Am. J. Geriatr. Psychiatry* 18 305–312. 10.1097/JGP.0b013e3181c2941b 20220585 PMC2865852

[B12] Bridges P. K. Jones M. T. (1968). Relationship of personality and physique to plasma cortisol levels in response to anxiety. *J. Neurol. Neurosurg. Psychiatry* 31 57–60. 10.1136/jnnp.31.1.57 5644931 PMC496286

[B13] Brunnstrom H. Passant U. Englund E. Gustafson L. (2013). History of depression prior to Alzheimer’s disease and vascular dementia verified post-mortem. *Arch. Gerontol. Geriatr.* 56 80–84. 10.1016/j.archger.2012.10.008 23116976

[B14] Burri A. Maercker A. Krammer S. Simmen-Janevska K. (2013). Childhood trauma and PTSD symptoms increase the risk of cognitive impairment in a sample of former indentured child laborers in old age. *PLoS One* 8:e57826. 10.1371/journal.pone.0057826 23469076 PMC3582641

[B15] Canet G. Chevallier N. Zussy C. Desrumaux C. Givalois L. (2018). Central role of glucocorticoid receptors in Alzheimer’s Disease and depression. *Front. Neurosci.* 12:739 10.3389/fnins.2018.00739 PMC6232776 30459541

[B16] Chopra S. Rathore A. Younas H. Pham L. V. Gu C. Beselman A. (2017). Obstructive sleep apnea dynamically increases nocturnal plasma free fatty acids, glucose, and cortisol during sleep. *J. Clin. Endocrinol. Metab.* 102 3172–3181. 10.1210/jc.2017-00619 28595341 PMC5587067

[B17] Comijs H. C. van den Kommer T. N. Minnaar R. W. Penninx B. W. Deeg D. J. (2011). Accumulated and differential effects of life events on cognitive decline in older persons: depending on depression, baseline cognition, or ApoE epsilon4 status? *J. Gerontol. B Psychol. Sci. Soc. Sci.* 66(Suppl. 1), i111–i120. 10.1093/geronb/gbr019 21422053

[B18] Copinschi G. Caufriez A. (2013). Sleep and hormonal changes in aging. *Endocrinol. Metab. Clin. North Am.* 42 371–389. 10.1016/j.ecl.2013.02.009 23702407

[B19] Cox S. R. MacPherson S. E. Ferguson K. J. Royle N. A. Maniega S. M. Hernandez Mdel C. (2015). Does white matter structure or hippocampal volume mediate associations between cortisol and cognitive ageing? *Psychoneuroendocrinology* 62 129–137. 10.1016/j.psyneuen.2015.08.005 26298692 PMC4642652

[B20] Csernansky J. G. Dong H. Fagan A. M. Wang L. Xiong C. Holtzman D. M. (2006). Plasma cortisol and progression of dementia in subjects with Alzheimer-type dementia. *Am. J. Psychiatry* 163 2164–2169. 10.1176/ajp.2006.163.12.2164 17151169 PMC1780275

[B21] Daskalakis N. P. Lehrner A. Yehuda R. (2013). Endocrine aspects of post-traumatic stress disorder and implications for diagnosis and treatment. *Endocrinol. Metab. Clin. North Am.* 42 503–513. 10.1016/j.ecl.2013.05.004 24011883

[B22] de Kloet E. R. Meijer O. C. de Nicola A. F. de Rijk R. H. Joels M. (2018). Importance of the brain corticosteroid receptor balance in metaplasticity, cognitive performance and neuro-inflammation. *Front. Neuroendocrinol.* 49:124–145. 10.1016/j.yfrne.2018.02.003 29428549

[B23] de Kloet E. R. Oitzl M. S. Joels M. (1999). Stress and cognition: are corticosteroids good or bad guys? *Trends Neurosci.* 22 422–426. 10.1016/S0166-2236(99)01438-1 10481183

[B24] Deeg D. J. Huizink A. C. Comijs H. C. Smid T. (2005). Disaster and associated changes in physical and mental health in older residents. *Eur. J. Public Health* 15 170–174. 10.1093/eurpub/cki126 15755778

[B25] Diniz B. S. Butters M. A. Albert S. M. Dew M. A. Reynolds C. F. III (2013). Late-life depression and risk of vascular dementia and Alzheimer’s disease: systematic review and meta-analysis of community-based cohort studies. *Br. J. Psychiatry* 202 329–335. 10.1192/bjp.bp.112.118307 23637108 PMC3640214

[B26] Domes G. Rothfischer J. Reichwald U. Hautzinger M. (2005). Inverted-U function between salivary cortisol and retrieval of verbal memory after hydrocortisone treatment. *Behav. Neurosci.* 119 512–517. 10.1037/0735-7044.119.2.512 15839797

[B27] Dong H. Csernansky J. G. (2009). Effects of stress and stress hormones on amyloid-beta protein and plaque deposition. *J. Alzheimers Dis.* 18 459–469. 10.3233/JAD-2009-1152 19584430 PMC2905685

[B28] Echouffo-Tcheugui J. B. Conner S. C. Himali J. J. Maillard P. DeCarli C. S. Beiser A. S. (2018). Circulating cortisol and cognitive and structural brain measures: the Framingham heart study. *Neurology* 91 e1961–e1970. 10.1212/WNL.0000000000006549 30355700 PMC6260201

[B29] Edwards K. M. Kamat R. Tomfohr L. M. Ancoli-Israel S. Dimsdale J. E. (2014). Obstructive sleep apnea and neurocognitive performance: the role of cortisol. *Sleep Med.* 15 27–32. 10.1016/j.sleep.2013.08.789 24269133 PMC3906433

[B30] Ennis G. E. An Y. Resnick S. M. Ferrucci L. O’Brien R. J. Moffat S. D. (2017). Long-term cortisol measures predict Alzheimer disease risk. *Neurology* 88 371–378. 10.1212/WNL.0000000000003537 27986873 PMC5272965

[B31] Ferguson E. (2008). Health anxiety moderates the daytime cortisol slope. *J. Psychosom. Res.* 64 487–494. 10.1016/j.jpsychores.2008.01.011 18440401

[B32] Flatt J. D. Gilsanz P. Quesenberry C. P. Jr. Albers K. B. Whitmer R. A. (2017). Post-traumatic stress disorder and risk of dementia among members of a health care delivery system. *Alzheimers Dement.* 14 28–34. 10.1016/j.jalz.2017.04.014 28627380 PMC5729063

[B33] Forget H. Lacroix A. Bourdeau I. Cohen H. (2016). Long-term cognitive effects of glucocorticoid excess in Cushing’s syndrome. *Psychoneuroendocrinology* 65 26–33. 10.1016/j.psyneuen.2015.11.020 26708069

[B34] Fountoulakis K. N. Pavlidis I. Tsolaki M. (2011). Life events and dementia: what is the nature of their relationship? *Psychiatry Res.* 190 156–158. 10.1016/j.psychres.2011.05.011 21621852

[B35] Fries E. Dettenborn L. Kirschbaum C. (2009). The cortisol awakening response (CAR): facts and future directions. *Int. J. Psychophysiol.* 72 67–73. 10.1016/j.ijpsycho.2008.03.014 18854200

[B36] Garcia-Banda G. Chellew K. Fornes J. Perez G. Servera M. Evans P. (2014). Neuroticism and cortisol: pinning down an expected effect. *Int. J. Psychophysiol.* 91 132–138. 10.1016/j.ijpsycho.2013.12.005 24361230

[B37] Geerlings M. I. Sigurdsson S. Eiriksdottir G. Garcia M. E. Harris T. B. Gudnason V. (2015). Salivary cortisol, brain volumes, and cognition in community-dwelling elderly without dementia. *Neurology* 85 976–983. 10.1212/WNL.0000000000001931 26291281 PMC4567466

[B38] Geerlings M. I. Sigurdsson S. Eiriksdottir G. Phillips C. Jonsson P. V. Gudnason V. (2017). Late-life depression, salivary cortisol, and incident dementia: the ages-REYKJAVIK study. *Alzheimers Dement.* 13:854 10.1016/j.jalz.2017.06.1207

[B39] Gerritsen L. Geerlings M. I. Bremmer M. A. Beekman A. T. Deeg D. J. Penninx B. W. (2009). Personality characteristics and hypothalamic-pituitary-adrenal axis regulation in older persons. *Am. J. Geriatr. Psychiatry* 17 1077–1084. 10.1097/JGP.0b013e3181bd1be6 20104064

[B40] Goodman Y. Bruce A. J. Cheng B. Mattson M. P. (1996). Estrogens attenuate and corticosterone exacerbates excitotoxicity, oxidative injury, and amyloid beta-peptide toxicity in hippocampal neurons. *J. Neurochem.* 66 1836–1844. 10.1046/j.1471-4159.1996.66051836.x 8780008

[B41] Green K. N. Billings L. M. Roozendaal B. McGaugh J. L. LaFerla F. M. (2006). Glucocorticoids increase amyloid-beta and tau pathology in a mouse model of Alzheimer’s disease. *J. Neurosci.* 26 9047–9056. 10.1523/JNEUROSCI.2797-06.2006 16943563 PMC6675335

[B42] Haba-Rubio J. Marti-Soler H. Tobback N. Andries D. Marques-Vidal P. Waeber G. (2017). Sleep characteristics and cognitive impairment in the general population: the HypnoLaus study. *Neurology* 88 463–469. 10.1212/WNL.0000000000003557 28039311

[B43] Haba-Rubio J. Ouanes S. Franc Y. Marques-Vidal P. Waeber G. Vollenweider P. (2018). Do diurnal cortisol levels mediate the association between sleep disturbances and cognitive impairment? *Neurobiol. Aging* 69 65–67. 10.1016/j.neurobiolaging.2018.05.001 29859364

[B44] Hamer M. O’Donnell K. Lahiri A. Steptoe A. (2010). Salivary cortisol responses to mental stress are associated with coronary artery calcification in healthy men and women. *Eur. Heart J.* 31 424–429. 10.1093/eurheartj/ehp386 19744954

[B45] Herbert J. Lucassen P. J. (2016). Depression as a risk factor for Alzheimer’s disease: genes, steroids, cytokines and neurogenesis - what do we need to know? *Front. Neuroendocrinol.* 41:153–171. 10.1016/j.yfrne.2015.12.001 26746105

[B46] Herrera-Guzman I. Gudayol-Ferre E. Herrera-Abarca J. E. Herrera-Guzman D. Montelongo-Pedraza P. Padros Blazquez F. (2010). Major depressive disorder in recovery and neuropsychological functioning: effects of selective serotonin reuptake inhibitor and dual inhibitor depression treatments on residual cognitive deficits in patients with major depressive disorder in recovery. *J. Affect. Disord.* 123 341–350. 10.1016/j.jad.2009.10.009 19896719

[B47] Hinkelmann K. Wingenfeld K. Kuehl L. K. Fleischer J. Heuser I. Wiedemann K. (2015). Stimulation of the mineralocorticoid receptor improves memory in young and elderly healthy individuals. *Neurobiol. Aging* 36 919–924. 10.1016/j.neurobiolaging.2014.09.008 25442112

[B48] Huang C. W. Lui C. C. Chang W. N. Lu C. H. Wang Y. L. Chang C. C. (2009). Elevated basal cortisol level predicts lower hippocampal volume and cognitive decline in Alzheimer’s disease. *J. Clin. Neurosci.* 16 1283–1286. 10.1016/j.jocn.2008.12.026 19570680

[B49] Joels M. (2006). Corticosteroid effects in the brain: U-shape it. *Trends Pharmacol. Sci.* 27 244–250. 10.1016/j.tips.2006.03.007 16584791

[B50] Johansson L. Guo X. Waern M. Ostling S. Gustafson D. Bengtsson C. (2010). Midlife psychological stress and risk of dementia: a 35-year longitudinal population study. *Brain* 133(Pt 8), 2217–2224. 10.1093/brain/awq116 20488887

[B51] Johansson L. Skoog I. Gustafson D. R. Olesen P. J. Waern M. Bengtsson C. (2012). Midlife psychological distress associated with late-life brain atrophy and white matter lesions: a 32-year population study of women. *Psychosom. Med.* 74 120–125. 10.1097/PSY.0b013e318246eb10 22286853

[B52] Jorm A. F. Mackinnon A. J. Christensen H. Henderson S. Scott R. Korten A. (1993). Cognitive functioning and neuroticism in an elderly community sample. *Pers. Individ. Differ.* 15 721–723. 10.1016/0191-8869(93)90013-S

[B53] Kim B. Feldman E. L. (2015). Insulin resistance as a key link for the increased risk of cognitive impairment in the metabolic syndrome. *Exp. Mol. Med.* 47:e149. 10.1038/emm.2015.3 25766618 PMC4351418

[B54] Kino T. Jaffe H. Amin N. D. Chakrabarti M. Zheng Y. L. Chrousos G. P. (2010). Cyclin-dependent kinase 5 modulates the transcriptional activity of the mineralocorticoid receptor and regulates expression of brain-derived neurotrophic factor. *Mol. Endocrinol.* 24 941–952. 10.1210/me.2009-0395 20357208 PMC2870940

[B55] Klaming R. Veltman D. J. Comijs H. C. (2016). The impact of personality on memory function in older adults-results from the longitudinal aging study Amsterdam. *Int. J. Geriatr. Psychiatry* 32 798–804. 10.1002/gps.4527 27329835

[B56] Kling M. A. Coleman V. H. Schulkin J. (2009). Glucocorticoid inhibition in the treatment of depression: can we think outside the endocrine hypothalamus? *Depress Anxiety* 26 641–649. 10.1002/da.20546 19133699 PMC3640810

[B57] Kulstad J. J. McMillan P. J. Leverenz J. B. Cook D. G. Green P. S. Peskind E. R. (2005). Effects of chronic glucocorticoid administration on insulin-degrading enzyme and amyloid-beta peptide in the aged macaque. *J. Neuropathol. Exp. Neurol.* 64 139–146. 10.1093/jnen/64.2.139 15751228

[B58] Lante F. Chafai M. Raymond E. F. Pereira A. R. Mouska X. Kootar S. (2015). Subchronic glucocorticoid receptor inhibition rescues early episodic memory and synaptic plasticity deficits in a mouse model of Alzheimer’s disease. *Neuropsychopharmacology* 40 1772–1781. 10.1038/npp.2015.25 25622751 PMC4915261

[B59] Lattanzi S. Silvestrini M. (2017). Letter re: long-term cortisol measures predict Alzheimer disease risk. *Neurology* 89:106. 10.1212/WNL.0000000000004074 28674159

[B60] LeBlanc J. Ducharme M. B. (2005). Influence of personality traits on plasma levels of cortisol and cholesterol. *Physiol. Behav.* 84 677–680. 10.1016/j.physbeh.2005.02.020 15885243

[B61] Lee B. K. Glass T. A. Wand G. S. McAtee M. J. Bandeen-Roche K. Bolla K. I. (2008). Apolipoprotein e genotype, cortisol, and cognitive function in community-dwelling older adults. *Am. J. Psychiatry* 165 1456–1464. 10.1176/appi.ajp.2008.07091532 18593777 PMC2579316

[B62] Lee C. M. Huxley R. R. Wildman R. P. Woodward M. (2008). Indices of abdominal obesity are better discriminators of cardiovascular risk factors than BMI: a meta-analysis. *J. Clin. Epidemiol.* 61 646–653. 10.1016/j.jclinepi.2007.08.012 18359190

[B63] Lehallier B. Essioux L. Gayan J. Alexandridis R. Nikolcheva T. Wyss-Coray T. (2016). Combined plasma and cerebrospinal fluid signature for the prediction of midterm progression from mild cognitive impairment to Alzheimer disease. *JAMA Neurol.* 73 203–212. 10.1001/jamaneurol.2015.3135 26659895 PMC5214993

[B64] Lesuis S. L. Weggen S. Baches S. Lucassen P. J. Krugers H. J. (2018). Targeting glucocorticoid receptors prevents the effects of early life stress on amyloid pathology and cognitive performance in APP/PS1 mice. *Transl. Psychiatry* 8:53. 10.1038/s41398-018-0101-2 29491368 PMC5830444

[B65] Lok A. Mocking R. J. Ruhe H. G. Visser I. Koeter M. W. Assies J. (2012). Longitudinal hypothalamic-pituitary-adrenal axis trait and state effects in recurrent depression. *Psychoneuroendocrinology* 37 892–902. 10.1016/j.psyneuen.2011.10.005 22094110

[B66] Lupien S. J. Maheu F. Tu M. Fiocco A. Schramek T. E. (2007). The effects of stress and stress hormones on human cognition: implications for the field of brain and cognition. *Brain Cogn.* 65 209–237. 10.1016/j.bandc.2007.02.007 17466428

[B67] Lupien S. J. McEwen B. S. (1997). The acute effects of corticosteroids on cognition: integration of animal and human model studies. *Brain Res. Brain Res. Rev.* 24 1–27. 10.1016/S0165-0173(97)00004-0 9233540

[B68] Lupien S. J. McEwen B. S. Gunnar M. R. Heim C. (2009). Effects of stress throughout the lifespan on the brain, behaviour and cognition. *Nat. Rev. Neurosci.* 10 434–445. 10.1038/nrn2639 19401723

[B69] Lupien S. J. Nair N. P. Briere S. Maheu F. Tu M. T. Lemay M. (1999). Increased cortisol levels and impaired cognition in human aging: implication for depression and dementia in later life. *Rev. Neurosci.* 10 117–139. 10.1515/REVNEURO.1999.10.2.117 10658955

[B70] Lupien S. J. Wilkinson C. W. Briere S. Ng Ying Kin N. M. Meaney M. J. (2002). Acute modulation of aged human memory by pharmacological manipulation of glucocorticoids. *J. Clin. Endocrinol. Metab.* 87 3798–3807. 10.1210/jcem.87.8.8760 12161513

[B71] Majer M. Nater U. M. Lin J. M. Capuron L. Reeves W. C. (2010). Association of childhood trauma with cognitive function in healthy adults: a pilot study. *BMC Neurol.* 10:61. 10.1186/1471-2377-10-61 20630071 PMC2910667

[B72] Martocchia A. Stefanelli M. Falaschi G. M. Toussan L. Ferri C. Falaschi P. (2016). Recent advances in the role of cortisol and metabolic syndrome in age-related degenerative diseases. *Aging Clin. Exp. Res.* 28 17–23. 10.1007/s40520-015-0353-0 25813987

[B73] McEwen B. S. (2007). Physiology and neurobiology of stress and adaptation: central role of the brain. *Physiol. Rev.* 87 873–904. 10.1152/physrev.00041.2006 17615391

[B74] McGaugh J. L. Roozendaal B. (2002). Role of adrenal stress hormones in forming lasting memories in the brain. *Curr. Opin. Neurobiol.* 12 205–210. 10.1016/S0959-4388(02)00306-9 12015238

[B75] Meier B. Perrig-Chiello P. Perrig W. (2002). Personality and memory in old age. *Aging Neuropsychol. Cogn.* 9 135–144. 10.1076/anec.9.2.135.9544

[B76] Meir Drexler S. Wolf O. T. (2016). The role of glucocorticoids in emotional memory reconsolidation. *Neurobiol. Learn. Mem.* 142(Pt A), 126–134. 10.1016/j.nlm.2016.11.008 27871996

[B77] Miller G. E. Chen E. Zhou E. S. (2007). If it goes up, must it come down? Chronic stress and the hypothalamic-pituitary-adrenocortical axis in humans. *Psychol. Bull.* 133 25–45. 10.1037/0033-2909.133.1.25 17201569

[B78] Miller G. E. Cohen S. Rabin B. S. Skoner D. P. Doyle W. J. (1999). Personality and tonic cardiovascular, neuroendocrine, and immune parameters. *Brain Behav. Immun.* 13 109–123. 10.1006/brbi.1998.0545 10373276

[B79] Miller K. G. Wright A. G. Peterson L. M. Kamarck T. W. Anderson B. A. Kirschbaum C. (2016). Trait positive and negative emotionality differentially associate with diurnal cortisol activity. *Psychoneuroendocrinology* 68 177–185. 10.1016/j.psyneuen.2016.03.004 26986092 PMC4864725

[B80] Nater U. M. Hoppmann C. Klumb P. L. (2010). Neuroticism and conscientiousness are associated with cortisol diurnal profiles in adults–role of positive and negative affect. *Psychoneuroendocrinology* 35 1573–1577. 10.1016/j.psyneuen.2010.02.017 20299157

[B81] Notarianni E. (2017). Cortisol: mediator of association between Alzheimer’s disease and diabetes mellitus? *Psychoneuroendocrinology* 81 129–137. 10.1016/j.psyneuen.2017.04.008 28458232

[B82] O’Banion M. K. (2013). It may take more than a shot: alternatives to immunotherapy for Alzheimer’s disease. *Biol. Psychiatry* 74 316–317. 10.1016/j.biopsych.2013.07.003 23932341

[B83] Ouanes S. Castelao E. Gebreab S. von Gunten A. Preisig M. Popp J. (2017a). Life events, salivary cortisol, and cognitive performance in nondemented subjects: a population-based study. *Neurobiol. Aging* 51 1–8. 10.1016/j.neurobiolaging.2016.11.014 28012996

[B84] Ouanes S. Castelao E. von Gunten A. Vidal P. M. Preisig M. Popp J. (2017b). Personality, cortisol, and cognition in non-demented elderly subjects: results from a population-based study. *Front. Aging Neurosci.* 9:63. 10.3389/fnagi.2017.00063 28352228 PMC5348534

[B85] Pedersen W. A. Wan R. Mattson M. P. (2001). Impact of aging on stress-responsive neuroendocrine systems. *Mech. Ageing Dev.* 122 963–983. 10.1016/S0047-6374(01)00250-0 11348661

[B86] Pietrzak R. H. Laws S. M. Lim Y. Y. Bender S. J. Porter T. Doecke J. (2017). Plasma cortisol, brain amyloid-beta, and cognitive decline in preclinical Alzheimer’s disease: a 6-year prospective cohort study. *Biol. Psychiatry Cogn. Neurosci. Neuroimaging* 2 45–52. 10.1016/j.bpsc.2016.08.006 29560886

[B87] Pineau F. Canet G. Desrumaux C. Hunt H. Chevallier N. Ollivier M. (2016). New selective glucocorticoid receptor modulators reverse amyloid-beta peptide-induced hippocampus toxicity. *Neurobiol. Aging* 45 109–122. 10.1016/j.neurobiolaging.2016.05.018 27459932

[B88] Pomara N. Doraiswamy P. M. Tun H. Ferris S. (2002). Mifepristone (RU 486) for Alzheimer’s disease. *Neurology* 58 1436–1436. 10.1212/wnl.58.9.1436 12011303

[B89] Popp J. (2013). Delirium and cognitive decline: more than a coincidence. *Curr. Opin. Neurol.* 26 634–639. 10.1097/WCO.0000000000000030 24152819

[B90] Popp J. Schaper K. Kolsch H. Cvetanovska G. Rommel F. Klingmuller D. (2009). CSF cortisol in Alzheimer’s disease and mild cognitive impairment. *Neurobiol. Aging* 30 498–500. 10.1016/j.neurobiolaging.2007.07.007 17716786

[B91] Popp J. Wolfsgruber S. Heuser I. Peters O. Hull M. Schroder J. (2015). Cerebrospinal fluid cortisol and clinical disease progression in MCI and dementia of Alzheimer’s type. *Neurobiol. Aging* 36 601–607. 10.1016/j.neurobiolaging.2014.10.031 25435336

[B92] Portella M. J. Harmer C. J. Flint J. Cowen P. Goodwin G. M. (2005). Enhanced early morning salivary cortisol in neuroticism. *Am. J. Psychiatry* 162 807–809. 10.1176/appi.ajp.162.4.807 15800161

[B93] Rock P. L. Roiser J. P. Riedel W. J. Blackwell A. D. (2014). Cognitive impairment in depression: a systematic review and meta-analysis. *Psychol. Med.* 44 2029–2040. 10.1017/S0033291713002535 24168753

[B94] Rothman S. M. Mattson M. P. (2010). Adverse stress, hippocampal networks, and Alzheimer’s disease. *Neuromol. Med.* 12 56–70. 10.1007/s12017-009-8107-9 19943124 PMC2833224

[B95] Saigh P. A. Yasik A. E. Oberfield R. A. Halamandaris P. V. Bremner J. D. (2006). The intellectual performance of traumatized children and adolescents with or without posttraumatic stress disorder. *J. Abnorm. Psychol.* 115 332–340. 10.1037/0021-843X.115.2.332 16737397 PMC3232057

[B96] Salvat-Pujol N. Labad J. Urretavizcaya M. de Arriba-Arnau A. Segalas C. Real E. (2017). Hypothalamic-pituitary-adrenal axis activity and cognition in major depression: the role of remission status. *Psychoneuroendocrinology* 76 38–48. 10.1016/j.psyneuen.2016.11.007 27883963

[B97] Sang Y. M. Wang L. J. Mao H. X. Lou X. Y. Zhu Y. J. (2018). The association of short-term memory and cognitive impairment with ghrelin, leptin, and cortisol levels in non-diabetic and diabetic elderly individuals. *Acta Diabetol.* 55 531–539. 10.1007/s00592-018-1111-5 29492658

[B98] Schaub C. Von Gunten A. Morin D. Wild P. Gomez P. Popp J. (2018). The effects of hand massage on stress and agitation among people with dementia in a hospital setting: a pilot study. *Appl. Psychophysiol. Biofeedback* 10.1007/s10484-018-9416-2 [Epub ahead of print]. 30209712 PMC6223738

[B99] Schommer N. C. Kudielka B. M. Hellhammer D. H. Kirschbaum C. (1999). No evidence for a close relationship between personality traits and circadian cortisol rhythm or a single cortisol stress response. *Psychol. Rep.* 84(3 Pt 1), 840–842. 10.2466/pr0.1999.84.3.840 10408206

[B100] Segerstrom S. C. Geiger P. J. Boggero I. A. Schmitt F. A. Sephton S. E. (2016). Endogenous cortisol exposure and declarative verbal memory: a longitudinal study of healthy older adults. *Psychosom. Med.* 78 182–191. 10.1097/PSY.0000000000000249 26569538 PMC4738083

[B101] Starkman M. N. Giordani B. Gebarski S. S. Berent S. Schork M. A. Schteingart D. E. (1999). Decrease in cortisol reverses human hippocampal atrophy following treatment of Cushing’s disease. *Biol. Psychiatry* 46 1595–1602. 10.1016/S0006-3223(99)00203-6 10624540

[B102] Stefanelli M. Martocchia A. De Marinis E. A. Falaschi G. M. Romano G. Rufo M. (2014). Treatment of insulin resistance in the neurodegeneration. *Recent Pat. CNS Drug Discov.* 9 54–63. 10.2174/1574889809666140410093006 24724584

[B103] Sudheimer K. D. O’Hara R. Spiegel D. Powers B. Kraemer H. C. Neri E. (2014). Cortisol, cytokines, and hippocampal volume interactions in the elderly. *Front. Aging Neurosci.* 6:153. 10.3389/fnagi.2014.00153 25071562 PMC4079951

[B104] Sundstrom A. Ronnlund M. Adolfsson R. Nilsson L. G. (2014). Stressful life events are not associated with the development of dementia. *Int. Psychogeriatr.* 26 147–154. 10.1017/S1041610213001804 24182362 PMC3854552

[B105] Suri D. Vaidya V. A. (2013). Glucocorticoid regulation of brain-derived neurotrophic factor: relevance to hippocampal structural and functional plasticity. *Neuroscience* 239 196–213. 10.1016/j.neuroscience.2012.08.065 22967840

[B106] Tatomir A. Micu C. Crivii C. (2014). The impact of stress and glucocorticoids on memory. *Clujul Med.* 87 3–6. 10.15386/cjm.2014.8872.871.at1cm2 26527987 PMC4462413

[B107] Tautvydaite D. Kukreja D. Antonietti J. P. Henry H. von Gunten A. Popp J. (2017). Interaction between personality traits and cerebrospinal fluid biomarkers of Alzheimer’s disease pathology modulates cognitive performance. *Alzheimers Res. Ther.* 9:6. 10.1186/s13195-017-0235-0 28153054 PMC5290611

[B108] Terracciano A. Stephan Y. Luchetti M. Albanese E. Sutin A. R. (2017). Personality traits and risk of cognitive impairment and dementia. *J. Psychiatr. Res.* 89 22–27. 10.1016/j.jpsychires.2017.01.011 28153642 PMC5374012

[B109] Terracciano A. Sutin A. R. An Y. O’Brien R. J. Ferrucci L. Zonderman A. B. (2014). Personality and risk of Alzheimer’s disease: new data and meta-analysis. *Alzheimers Dement.* 10 179–186. 10.1016/j.jalz.2013.03.002 23706517 PMC3783589

[B110] Tiemensma J. Andela C. D. Biermasz N. R. Romijn J. A. Pereira A. M. (2016). Mild cognitive deficits in patients with primary adrenal insufficiency. *Psychoneuroendocrinology* 63 170–177. 10.1016/j.psyneuen.2015.09.029 26454105

[B111] Toledo J. B. Toledo E. Weiner M. W. Jack C. R. Jr. Jagust W. Lee V. M. (2012). Cardiovascular risk factors, cortisol, and amyloid-beta deposition in Alzheimer’s disease neuroimaging initiative. *Alzheimers Dement.* 8 483–489. 10.1016/j.jalz.2011.08.008 23102118 PMC3668456

[B112] van Eck M. Berkhof H. Nicolson N. Sulon J. (1996). The effects of perceived stress, traits, mood states, and stressful daily events on salivary cortisol. *Psychosom. Med.* 58 447–458. 10.1097/00006842-199609000-00007 8902896

[B113] van Gelder B. M. Tijhuis M. Kalmijn S. Giampaoli S. Nissinen A. Kromhout D. (2006). Marital status and living situation during a 5-year period are associated with a subsequent 10-year cognitive decline in older men: the FINE study. *J. Gerontol. B Psychol. Sci. Soc. Sci.* 61 213–219. 10.1093/geronb/61.4.P213 16855033

[B114] Vogel S. Fernandez G. Joels M. Schwabe L. (2016). Cognitive adaptation under stress: a case for the mineralocorticoid receptor. *Trends Cogn. Sci.* 20 192–203. 10.1016/j.tics.2015.12.003 26803208

[B115] VonDras D. D. Powless M. R. Olson A. K. Wheeler D. Snudden A. L. (2005). Differential effects of everyday stress on the episodic memory test performances of young, mid-life, and older adults. *Aging Ment. Health* 9 60–70. 10.1080/13607860412331323782 15841833

[B116] Ward L. Mathias J. L. Hitchings S. E. (2007). Relationships between bereavement and cognitive functioning in older adults. *Gerontology* 53 362–372. 10.1159/000104787 17622731

[B117] Webster S. P. McBride A. Binnie M. Sooy K. Seckl J. R. Andrew R. (2017). Selection and early clinical evaluation of the brain-penetrant 11beta-hydroxysteroid dehydrogenase type 1 (11beta-HSD1) inhibitor UE2343 (Xanamem). *Br. J. Pharmacol.* 174 396–408. 10.1111/bph.13699 28012176 PMC5301048

[B118] Wolkowitz O. M. Burke H. Epel E. S. Reus V. I. (2009). Glucocorticoids. Mood, memory, and mechanisms. *Ann. N. Y. Acad. Sci.* 1179 19–40. 10.1111/j.1749-6632.2009.04980.x 19906230

[B119] Wolkowitz O. M. Reus V. I. (1999). Treatment of depression with antiglucocorticoid drugs. *Psychosom. Med.* 61 698–711. 10.1097/00006842-199909000-00011 10511017

[B120] Woods D. L. Beck C. Sinha K. (2009). The effect of therapeutic touch on behavioral symptoms and cortisol in persons with dementia. *Forsch. Komplementmed.* 16 181–189. 10.1159/000220479 19657203

[B121] Xavier F. M. Ferraz M. P. Trentini C. M. Freitas N. K. Moriguchi E. H. (2002). Bereavement-related cognitive impairment in an oldest-old community-dwelling Brazilian sample. *J. Clin. Exp. Neuropsychol.* 24 294–301. 10.1076/jcen.24.3.294.983 11992212

[B122] Yoshino A. Kimura Y. Yoshida T. Takahashi Y. Nomura S. (2005). Relationships between temperament dimensions in personality and unconscious emotional responses. *Biol. Psychiatry* 57 1–6. 10.1016/j.biopsych.2004.09.027 15607293

[B123] Zhang B. Zhang Y. Xu T. Yin Y. Huang R. Wang Y. (2017). Chronic dexamethasone treatment results in hippocampal neurons injury due to activate NLRP1 inflammasome in vitro. *Int. Immunopharmacol.* 49 222–230. 10.1016/j.intimp.2017.05.039 28605710

[B124] Zverova M. Fisar Z. Jirak R. Kitzlerova E. Hroudova J. Raboch J. (2013). Plasma cortisol in Alzheimer’s disease with or without depressive symptoms. *Med. Sci. Monit.* 19 681–689. 10.12659/MSM.889110 23955525 PMC3751335

